# Is wearing a face mask associated with symptomatic dry eye disease among medical students during the COVID-19 era? An online survey

**DOI:** 10.1186/s12886-022-02377-z

**Published:** 2022-04-07

**Authors:** Wedad Al-dolat, Luai Abu-Ismail, Almu’atasim Khamees, Noor Alqudah, Mohammed M. Abukawan, Hamzeh Mohammad Alrawashdeh, Waleed Al Momani, Khaled A. Kheirallah

**Affiliations:** 1 Department of Ophthalmology, Faculty of Medicine, Yarmouk University, Irbid, Jordan; 2 Department of Clinical Medical Sciences, Faculty of Medicine, Yarmouk University, Irbid, Jordan; 3 Department of Ophthalmology, Jordan University of Science and Technology, Irbid, Jordan; 4 Faculty of Medicine, Herbal Dynasty Medical Center, Mutah University, Amman, Jordan; 5 Sharif Eye Centers, Amman, Jordan; 6 Department of Basic Medical Sciences, Faculty of Medicine, Yarmouk University, Irbid, Jordan; 7 Department of Public Health, Medical School of Jordan University of Science and Technology, P.O. Box 3020, 22110 Irbid, Jordan

**Keywords:** Dry eye, Facemask, DED, COVID-19, Medical students, Jordan

## Abstract

**Background:**

Coronavirus disease 2019 has necessitate the routine use of masks worldwide. This study assessed the relationship between wearing a facemask and dry eye disease (DED) among a sample of medical students in Jordan.

**Methods:**

This cross-sectional online survey enrolled medical students from all medical schools in Jordan. The questionnaire, which was shared via social media platforms, assessed sociodemographic information, ocular and medical history, facemask-wearing habits, the use of ocular devices, and the relationship with ocular discomfort. The ocular surface disease index (OSDI) questionnaire was also administered to quantify DED symptoms.

**Results:**

A total of 1,219 students participated in this study. In total, 58.3% participants were females, and 52% were in the clinical science years. Symptomatic DED was found in 71.7% of participants. Female sex, basic science years, allergy reporting, and spending more than 6 h looking at screens were significantly associated with symptomatic DED.

**Conclusion:**

Wearing a facemask was not significantly associated with symptomatic DED. Further studies are needed to investigate the effect of wearing a facemask on the ocular surface.

**Supplementary Information:**

The online version contains supplementary material available at 10.1186/s12886-022-02377-z.

## Background

A new global pandemic emerged in December 2019 that was caused by a new coronavirus strain called severe acute respiratory syndrome coronavirus-2 (SARS-CoV-2). The recent coronavirus disease (COVID-19) pandemic was associated with increased morbidity and mortality and has impacted the lives of the global population. SARS-CoV-2 is highly contagious and easily transmitted from human to human via respiratory droplets and direct contact, resulting in an enormous number of infected individuals [1].

SARS-CoV-2 has had an obvious impact on eye health through direct and indirect mechanisms. Since the beginning of the outbreak in December 2019, several published studies suggested that SARS-CoV-2 could cause conjunctivitis [2–4], whereas other studies described the presence of dry eye symptoms in patients with COVID-19 [3–5]. The public health measures that have been implemented, such as the mass use of facemasks, potentially have an indirect impact on the health of the ocular surface by causing or increasing ocular irritation and dryness [6–8].

Facemasks are used for the protection of healthy persons (worn to protect oneself when in contact with an infected individual) and as a method of source control (worn by an infected individual to prevent onwards transmission) [9]. The World Health Organization advised people to follow strict guidelines to protect themselves, such as improving hand hygiene, wearing facemasks and avoiding gatherings as much as possible [10]. In Jordan, the government issued an order requiring all individuals to wear facemasks prior to entering all public spaces and maintaining social distancing in those entities [11]. Huge gatherings, such as university classes, were restricted across the country, and E-learning practices were employed. Therefore, on August 25, 2020, the government announced that medical students enrolled in the basic sciences years (years 1 to 3) should continue to use E-learning, whereas students in their clinical science years (years 4–6) were allowed to attend their clinical training at hospitals provided they follow very strict rules on infection control to protect them from being infected with SARS-CoV-2 and control the spread of the disease [12].

Wearing facemasks was an essential measure of control during the COVID-19 pandemic. However, during recent periods of face mask utilization, a corresponding increase in ocular irritation and dryness was observed in individuals who never previously suffered from dry eyes [6–8], and air blowing upwards from behind the mask into the eyes has been implicated in accelerating the evaporation and instability of the tear film [13, 14].

Dry eye disease (DED) is a multifactorial disease involving tears and the ocular surface and is characterized by homeostatic disturbances of the ocular surface and tear film [15]. Dry eye is clinically subdivided into two subtypes: aqueous-deficient DED (with decreased tear secretion) and hyperevaporative DED (with increased tear evaporation) [15].

Medical students are at risk of DED due to the excessive reliance on electronic devices for e-learning and possibly the prolonged use of facemasks. The aim of this study was to assess the relationship between the wearing of facemasks and the presence of DED among medical students in Jordan during the COVID-19 pandemic.

## Methodology

### Sample and data collection

A descriptive cross-sectional study design was used. The study population consisted of medical students from all six medical schools in Jordan during all years of study (years 1–6). The study utilized an online questionnaire written in English and delivered to the students between October 26, 2020 and November 4, 2020 (Supplementary file 1).

The online questionnaire was created using Google Forms, and the invitation to participate in the study was posted on several online platforms at each medical school. Class representatives for each academic year were involved in distributing the questionnaire link to students directly. The invitation to participate was distributed to approximately 5,000 medical students.

The questionnaire contained 18 closed-ended questions distributed over three main sections: 1. Sociodemographic information, 2. Ocular and medical history, and 3. Information on facemask wearing and the use of electronic devices and the relationship with ocular discomfort. The ocular surface index questionnaire (OSDI) [16], which is designed to provide a rapid assessment of symptoms related to ocular irritation and dry eye disease during the previous week, was added at the end of the questionnaire (Supplementary file 2).

The questionnaire was piloted before the start of the study (*n* = 15 students), and proper modifications were made. The questionnaire was reviewed by expert ophthalmologists for relevance, simplicity, and internal consistency. Participation in the study was voluntary. In addition, personal identifiers were not collected, and a consent statement regarding voluntary participation in the study was included at the beginning of the questionnaire. The study was conducted according to the Helsinki Declaration principles and was approved by the Institutional Review Board of the Jordan University of Science and Technology.

### Statistical analysis

Data were summarized as frequencies and proportions. The chi-square test was used to analyse differences between categorical variables. Logistic regression was used to analyse factors associated with dry eye disease. Statistical significance was considered at a *P* value of < 0.05. Statistical analysis was performed using the Statistical Program for Social Sciences (IBM SPSS Corp, SPSS Statistics ver. 25, USA).

## Results

A total of 1219 students from six medical faculties in Jordan responded and completed the questionnaire, representing a response rate of 24.4%. Overall, 711 (58.3%) of respondents were females, and 634 (52%) were in the clinical science years. Only 175 (14.4%) used contact lenses, and nearly two-thirds of students reported wearing eyeglasses (761, 62.4%). Greater than one-third (475, 39%) of the students reported using eye lubricants with variable frequencies, 40 (3.3%) students reported having laser vision correction surgery performed, and 293 (24.0%) students reported having allergies. The demographic characteristics of the study participants are presented in Table 1. Most students (1114, 91.4%) reported increased use of screens and electronic devices during the COVID-19 pandemic, and 901 students (73.9%) reported spending more than 6 h daily looking at the screens during the last few months (Table 1).

**Table 1:** Demographic characteristics of the study participants

**Variable**	**Number (%)**
**Sex**	
Male	508 (41.7%)
Female	711 (58.3%)
**Year of study in Medical School**	
Basic Science Year	585 (48.0%)
Clinical Science Year	634 (52.0%)
**Contact lenses users**	
Always	15 (1.2%)
Most of the time	35 (2.9%)
Sometimes	125 (10.3%)
Never	1044 (85.6%)
**Wearing eyeglasses**	
I do not wear	458 (37.6%)
Regularly	521 (42.7%)
Sometimes	240 (19.7%)
**Laser vision correction surgery (LASIK or laser)**	
No	1179 (96.7%)
Less than 6 months ago	14 (1.1%)
More than 6 months ago	26 (2.1%)
**Taking any medication**	
No	923 (75.7%)
Allergy medication	91 (7.5%)
Acne medication	54 (4.4%)
Medication for another disease	151 (12.4%)
**Do you have any allergies**	
No	926 (76.0%)
Yes	293 (24.0%)
**Using eye lubricant**	
1 to 3 times/day	86 (7.1%)
More than three times a day	37 (3.0%)
Only when I need to	352 (28.9%)
No, I do not use	744 (61.0%)
**Do you think that you used screens more during the COVID-19 pandemic?**	
Yes	1114 (91.4%)
No	105 (8.6%)
**Select the amount of time spent looking at the screens**	
Less than 2 h/day	18 (1.5%)
2–4 h/day	54 (4.4%)
4–6 h/day	246 (20.2%)
More than 6 h/day	901 (37.9%)

Regarding the use of face masks, 546 (44.8%) students reported wearing a face mask at least 3 h a day, and student who wore face masks were significantly more likely to be in their clinical science years (*P* < 0.001). A total of 733 (60.1%) students reported wearing properly fitting face masks (to cover the mouth and nose) all the time, whereas only 79 (6.5%) reported not wearing a properly fitting mask at all. New eye discomforts, including symptoms of dryness (photophobia, foreign body sensation or burning) caused by wearing the facemask, was reported by 272 (22.3%) students. Among students already suffering from dry eye, 304 (32.7%) stated that wearing facemasks worsened their condition. Most eyeglasses wearers (592, 77.8%) reported that wearing facemasks was annoying due to eyeglass fogging (Table 2).

**Table 2:** Facemask wear and eye discomfort symptoms

Question		Number (%)
Average hours/day of wearing a facemask	More than 6 h/day	84 (6.9%)
	More than 3 and less than 6 h a day	462 (37.9%)
	From 1–3 h/day	522 (42.8%)
	Less than 1 h/day	151 (12.4%)
Fit the mask to cover mouth and nose	Always	733 (60.1%)
	Most of the time	317 (26%)
	Sometimes	90 (7.4%)
	I do not fit the mask	79 (6.5%)
Has wearing a facemask caused any new eye discomfort: symptoms of dryness, such as photophobia, foreign body sensation, or burning?	Yes	272 (22.3%)
	No	700 (57.4%)
	Neutral	247 (20.3%)
If you already suffer from dry eyes, has wearing a facemask caused the condition to become worse?	Much worse	22 (1.8%)
	Moderately worse	82 (6.7%)
	Slightly worse	200 (16.4%)
	I do not have dryness	288 (23.6%)
	No	627 (51.4%)
Does wearing a facemask with the eyeglasses hamper your vision, e.g., cause fogging?	Yes	592 (48.6%)
	No	169 (13.9%)
	I do not wear eyeglasses	458 (37.6%)

A total of 874 (71.7%) students were considered to have dry eye disease according to the OSDI. The presence of DED was significantly associated with female sex (*P* < 0.001), wearing a facemask for 3 h and less a day (*P* = 0.048), being in the basic science years at medical school (*P* < 0.001), spending more than 6 h looking at screens and electronic devices (*P* < 0.001) and having a history of allergies (*P* < 0.001) (Table 3).

**Table 3:** OSDI in relation to other variables

	**OSDI Number (%)**					***P*-value**
	**Normal**	**Mild**	**Moderate**	**Severe**	**Total**	
Sex						
Female	143 (11.7%)	110 (9.0%)	101 (8.3%)	357 (29.3%)	711 (58.3%)	< 0.001
Male	202 (16.6%)	97 (8.0%)	69 (5.7%)	140 (11.5%)	508 (41.7%)	
Year of study in Medical School						
Basic Science	113 (9.3%)	85 (7.0%)	84 (6.9%)	303 (24.9%)	585 (48.0%)	< 0.001
Clinical	232 (19.0%)	122 (10.0%)	86 (7.1%)	194 (15.9%)	634 (52.0%)	
Average hours/day of wearing face mask						
More than 6 h/day	20 (1.6%)	15 (1.2%)	15 (1.2%)	34 (2.8%)	84 (6.9%)	0.048
More than 3 and less than 6 h/day	150 (12.3%)	91 (7.5%)	59 (4.8%)	162 (13.3%)	462 (37.9%)	
From 1–3 h/day	145 (11.9%)	80 (6.6%)	69 (5.7%)	228 (18.7%)	522 (42.8%)	
Less than 1 h/day	30 (2.5%)	21 (1.7%)	27 (2.2%)	73 (6.0%)	151 (12.4%)	
Time spent looking at screens (mobile, laptop, iPad, or any electronic device) per day						
Less than 2 h/day	2 (0.2%)	2 (0.2%)	0 (0.0%)	14 (1.1%)	18 (1.5%)	< 0.001
2–4 h/day	23 (1.9%)	9 (0.7%)	6 (0.5%)	16 (1.3%)	54 (4.4%)	
4–6 h/day	93 (7.6%)	50 (4.1%)	32 (2.6%)	71 (5.8%)	246 (20.2%)	
More than 6 h/day	227 (18.6%)	146 (12.0%)	132 (10.8%)	396 (32.5%)	901 (73.9%)	
History of any allergies						
Yes	56 (4.6%)	45 (3.7%)	39 (3.2%)	153 (12.6%)	293 (24.0%)	< 0.001
No	289 (23.7%)	162 (13.3%)	131 (10.7%)	344 (28.2%)	926 (76.0%)	
Total	345 (28.3%)	207 (17%.0)	170 (13.9%)	497 (40.8%)	1219 (100.0%)	

Multivariate analysis using stepwise binary logistic regression was performed involving all variables with a *P* value ≤ 0.25 in the cross tabulation analysis to identify independent variables associated with DED. Female students were at two-fold greater risk of having eye dryness (OR = 2.4, *P*-value < 0.001). Interestingly, wearing facemasks was not associated with DED (Table 4).

**Table 4:** Multivariate logistic regression analysis of factors associated with symptomatic DED

**Factor**	**Odds Ratio**	**95% C.I**		***P* value**
		**Lower**	**Upper**	
**Sex**				
Male^a^	Reference			
Female	2.4	1.84	3.12	< 0.001
**Study level**				
Clinical Science^a^	Reference			
Basic Science	2.2	1.66	2.86	< 0.001
**Allergies**				
None^a^	Reference			
Yes	1.8	1.28	2.50	0.001
**Time using electronic devices/day**				
≤ 6 hours^a^	Reference			
> 6 h	1.4	1.01	1.77	0.040

^a^Reference category

## Discussion

There has been a recent awareness of mask-associated dry eye symptoms due to ill fitted masks and leakage of air causing increased evaporation on the surface of the eye. Many studies have suggested that air conviction around the eyes affects the ocular surface. For example, in patients with OSA syndrome who use continuous positive airway pressure (CPAP) masks, CPAP therapy increases ocular irritation and tear evaporation due to air escaping from the mask [17, 18]. Some studies have described that taping the upper mask edge blocks exhaled air from directly entering the eyes and thus results in significantly better ocular surface stability [13, 19]. In this study, we aimed to assess the relationship between facemask wearing and DED.

Recent studies reported a marked increase in dry eye symptoms among regular mask users, including individuals who have never suffered from dry eyes [6, 7]. Other studies showed that tear film stability in terms of tear breakup time (TBUT) was significantly reduced after prolonged mask use for more than 8 h [13, 20]. In a case report by Chadwick, a 66-year-old patient who had uneventful cataract surgery developed severe exposure keratopathy that significantly improved after minimizing mask wear and treating the exposure keratopathy with a gauze pad [21]. Giannaccare et al. found that a significant proportion of their studied population experienced the onset of ocular discomfort symptoms that required eye lubrication during the COVID-19 pandemic [7]. They identified two main mechanisms that could be responsible for the onset or worsening of dry eye symptoms during the pandemic: increased use of video display terminals (VDT) and wearing face masks [7]. In a recent study, Boccardo [8] found that among their studied population, 18.3% experienced mask-associated dry eye (MADE). In that study, MADE was defined as a condition in which dry eye symptoms were present at least occasionally and became worse with the use of a facemask [8]. However, in our study, approximately one-quarter of students said that they experienced new eye symptoms, such as symptoms of dryness with mask wear. Another one-quarter of students reported exacerbation of dry eye symptoms with mask wearing. However, after performing logistic regression to identify independent variables associated with DED results from OSDI, no significant relationship between wearing a mask and symptomatic DED was noted. In this study, we relied on self-reported responses regarding dry eye symptoms in the context of wearing a mask, hours spent wearing a mask, and proper mask fit. Eye dryness, mask wearing and proper mask fit were not directly assessed in students. We think our results can be explained by the lack of commitment to infectious control strategies, including wearing a mask. Therefore, whether students were committed to proper mask wearing and if they wore the mask correctly is not clear. In September 2020, an audit was performed in Jordan to reveal the levels of a general commitment to infectious control strategies in various sectors, and the results were shocking and disappointing given the general low commitment [22]. In the literature, another study found that subjects who wore facemasks for more than 6 h a day did not have higher OSDI scores than those who wore masks for 3 to 6 h [23]. This result was attributed to the fact that the group who wore the mask for more than 6 h included healthy, young individuals without previous DED [23].

Strikingly, this study showed a high prevalence of symptomatic DED (71.7%) among medical students. Similarly, Hyon et al. reported a high prevalence of dry eye symptoms (27.1%) among undergraduate medical students that was significantly associated with female sex, contact lens wear, prolonged computer use and high stress scores [24]. Another study from Ghana also showed a high prevalence of DED symptoms (44.3%) among university students [25].

Studies worldwide have shown that dry eye is more common in females than males [15, 26]. A study reported that the effect of sex on the prevalence of DED is common in individuals older than 40 years of age [15]. In this study, DED symptoms were 2.4 more common in females than males, and the age of the studied population ranged from 18 to 24 years. This finding is comparable with the results of a recent study from the Netherlands that revealed that the prevalence of dry eye symptoms was higher in females than males across all age groups but was more prominent in older individuals [27].

In this study, those who spent more than 6 h looking at the screens were 1.4 times more likely to have DED than those who spent less than 6 h. Another study reported the effect of VDT on tear film stability and demonstrated that DED is directly proportionate to exposure time to VDT [28]. This finding might explain why basic science students were 2.2 times more likely to have DED than clinical students. At the time of our study, basic science students were learning from home by E-learning; thus, the finding that these students were spending more time looking at screens was expected. This finding is similar to another study that showed that students who followed their classes online reported that they experienced all of the symptoms associated with DED more frequently than students attending school in person, and the difference was statistically significant [29]. Students with any self-reported allergy were 1.8 times more likely to have symptomatic DED. Asiedu et al. also reported that university students with any allergy were more likely to have symptomatic DED [25].

One of limitations of the present study is that the study did not correlate the presence of dry eye symptoms with objective clinical tests. It was reported that current dry eye tests show a weak association with disease symptomatology [30]. Further work is needed to correlate the disease signs with the reported symptoms in mask wearers. Another limitation is that we assessed mask wearing using a questionnaire, and we did not directly observe how medical students fitted the mask and whether they wore it correctly. The subjective nature of the OSDI represents another limitation.

## Conclusion

In this study, we did not find a significant relationship between wearing face masks and DED among medical students. Whether mask wear is responsible for the onset or worsening of DED symptoms remains to be elucidated. Moreover, more studies are needed to reveal the effect of wearing a mask on the ocular surface and tear film stability.

## Supplementary Information

**Additional file 1.**

**Additional file 2.**

## Data Availability

The datasets used and/or analysed during the current study are available from the corresponding author on reasonable request.

## Abbreviations

DED: Dry eye disease
COVID-19: Coronavirus disease 2019
OSDI: Ocular surface disease index
TBUT: Tear break-up time
OR: Odds ratio
CI: Confidence interval
SARS-COV2: Severe acute respiratory syndrome-coronavirus 2
OSA: Obstructive sleep apnea
CPAP: Continuous positive airway pressure
MADE: Mask-associated dry eye
VDT: Visual display terminal

## References

[1] Cai J, Sun W, Huang J, Gamber M, Wu J, He G (2020). Indirect Virus Transmission in Cluster of COVID-19 Cases, Wenzhou, China, 2020. Emerg Infect Dis.

[2] Chen L, Liu M, Zhang Z (2020). Ocular manifestations of a hospitalised patient with confirmed 2019 novel coronavirus disease. Br J Ophthalmol.

[3] Wu P, Duan F, Luo C (2020). Characteristics of Ocular Findings of Patients With Coronavirus Disease 2019 (COVID-19) in Hubei Province, China. JAMA Ophthalmology.

[4] Yu Jun IS, Anderson DE, Zheng Kang AE, et al. Assessing Viral Shedding and Infectivity of Tears in Coronavirus Disease 2019 (COVID-19) Patients. Ophthalmology. Published online March 2020:S0161642020303110. doi:10.1016/j.ophtha.2020.03.026 10.1016/j.ophtha.2020.03.026 PMC7151491 32291098

[5] Hong N, Yu W, Xia J, Shen Y, Yap M, Han W (2020). Evaluation of ocular symptoms and tropism of SARS-CoV-2 in patients confirmed with COVID-19. Acta Ophthalmol.

[6] Moshirfar M, West WB, Marx DP (2020). Face Mask-Associated Ocular Irritation and Dryness. Ophthalmol Therapy.

[7] Giannaccare G, Vaccaro S, Mancini A, Scorcia V (2020). Dry eye in the COVID-19 era: how the measures for controlling pandemic might harm ocular surface. Graefe’s Arch Clin Exp Ophthalmol.

[8] Boccardo L. Self-reported symptoms of mask-associated dry eye: A survey study of 3,605 people. Contact Lens and Anterior Eye. 2021;0(0). doi:10.1016/j.clae.2021.01.003 10.1016/j.clae.2021.01.003 PMC7816875 33485805

[9] Advice on the use of masks in the context of COVID-19: interim guidance, 5 June 2020. Published 2020. https://apps.who.int/iris/handle/10665/332293. Accessed 1 Jan 2021.

[10] Advice for the public on COVID-19 – World Health Organization. https://www.who.int/emergencies/diseases/novel-coronavirus-2019/advice-for-public. Accessed 1 Jan 2021.

[11] Jordan News Agency (Petra). https://www.petra.gov.jo/Include/InnerPage.jsp?ID=24863&lang=en&name=en_news Accessed 4 Jan 2021.

[12] MOHE_News - 25/8/2020. http://www.mohe.gov.jo/ar/Lists/MOHE_News/Display.aspx?ID=1035. Accessed 1 Jan.

[13] Aksoy M, Simsek M (2021). Evaluation of Ocular Surface and Dry Eye Symptoms in Face Mask Users. Eye Contact Lens.

[14] Arriola-Villalobos P, Burgos-Blasco B, Vidal-Villegas B, Oribio-Quinto C, Ariño-Gutiérrez M, Diaz-Valle D, Benitez-del-Castillo JM (2021). Effect of Face Mask on Tear Film Stability in Eyes With Moderate-to-Severe Dry Eye Disease. Cornea.

[15] Craig JP, Nichols KK, Akpek EK (2017). TFOS DEWS II Definition and Classification Report. Ocul Surf.

[16] Walt J, Rowe M, Stern K (1997). Evaluating the functional impact of dry eye: the Ocular Surface Disease Index. Drug Inf J.

[17] Salinas R, Puig M, Fry CL, Johnson DA, Kheirkhah A (2020). Floppy eyelid syndrome: A comprehensive review. Ocul Surf.

[18] Hayirci E, Yagci A, Palamar M, Basoglu OK, Veral A (2012). The effect of continuous positive airway pressure treatment for obstructive sleep apnea syndrome on the ocular surface. Cornea.

[19] Nair S, Kaur M, Sah R, Titiyal JS. Impact of Taping the Upper Mask Edge on Ocular Surface Stability and Dry Eye Symptoms [published online ahead of print, 2022 Jan 13]. Am J Ophthalmol. 2022;S0002–9394(22)00007–1. doi:10.1016/j.ajo.2022.01.006 10.1016/j.ajo.2022.01.006 35038414

[20] Esen Baris M, Guven Yilmaz S, Palamar M. Impact of prolonged face mask wearing on tear break-up time and dry eye symptoms in health care professionals [published online ahead of print, 2022 Feb 4]. Int Ophthalmol. 2022;1–4. doi:10.1007/s10792-022-02213-9 10.1007/s10792-022-02213-9 PMC8815392 35119609

[21] Chadwick O, Lockington D (2021). Addressing post-operative Mask-Associated Dry Eye (MADE). Eye.

[22] Ipsos, (2020) Compliance with Health & Safety standards in Jordan, Ipsos. Available from: https://www.ipsos.com/en-jo/compliance-health-safety-standards-jordan. Accessed 8 Mar 2021.

[23] Krolo I, Blazeka M, Merdzo I, Vrtar I, Sabol I, Petric-Vickovic I (2021). Mask-Associated Dry Eye During COVID-19 Pandemic-How Face Masks Contribute to Dry Eye Disease Symptoms. Med Arch.

[24] Hyon JY, Yang HK, Han SB (2019). Dry Eye Symptoms May Have Association With Psychological Stress in Medical Students. Eye Contact Lens.

[25] Asiedu K, Kyei S, Boampong F, Ocansey S (2017). Symptomatic Dry Eye and Its Associated Factors: A Study of University Undergraduate Students in Ghana. Eye Contact Lens.

[26] Vehof J, Sillevis Smitt-Kamminga N, Nibourg SA, Hammond CJ (2018). Sex differences in clinical characteristics of dry eye disease. Ocul Surf.

[27] Vehof J, Snieder H, Jansonius N, Hammond CJ. Prevalence and risk factors of dry eye in 79,866 participants of the population-based Lifelines cohort study in the Netherlands [published online ahead of print, 2020 May 4]. The Ocular Surface. 2020;S1542–0124(20)30069–0. doi:10.1016/j.jtos.2020.04.00521. 10.1016/j.jtos.2020.04.005 32376389

[28] Mehra D, Galor A (2020). Digital Screen Use and Dry Eye: A Review. Asia Pac J Ophthalmol.

[29] García-Ayuso D, Di Pierdomenico J, Moya-Rodríguez E, Valiente-Soriano FJ, Galindo-Romero C, Sobrado-Calvo P (2021). Assessment of dry eye symptoms among university students during the COVID-19 pandemic. Clin Exp Optom.

[30] Song H, Zhang M, Hu X (2017). Correlation Analysis of Ocular Symptoms and Signs in Patients with Dry Eye. J Ophthalmol.

