# Supplementary material for: Is wearing a face mask associated with symptomatic dry eye disease among medical students during the COVID-19 era? An online survey
Source: BMC Ophthalmol. 2022 Apr 7;22:159. doi: 10.1186/s12886-022-02377-z (PMC8988476; doi:10.1186/s12886-022-02377-z)
Supplement: Supplementary file 1 — Additional file 1. [file 12886_2022_2377_MOESM1_ESM.docx]

1. **What is your sex?**

Male

Female

1. **year of study in Medical school :** 1 2 3 4 5 6
2. **Do you wear contact lenses?**

Never

Sometimes

Most of the time

Always

1. **Do you wear eyeglasses?**

I do not wear eyeglasses

Regularly

Sometimes

**5. Have you had refractive surgery like LASIK or Laser?**

No

Less than 6 months ago

More than 6 months ago

1. **Do you take any medication:**

No

Allergy medication

Acne medication

Medication for another disease

1. **Do you have any allergies?**

No

Yes

1. **Do you use eye lubricant?**

1 to 3 times/day

More than three times a day

Only when I need to

No, I do not use

1. **How often do you wear the face mask during the day on average?**

More than 6 hours/day

More than 3 and less than 6 hours a day

From 1-3 hours/day

Less than 1 hour/day

1. **Do you fit your mask properly covering the mouth and nose?**

Always

Most of the time

Sometimes

I do not fit the mask

1. **Has wearing the face mask caused any new eye discomfort: ie symptoms of dryness like photophobia, foreign body sensation or burning?**

Yes

No

Neutral

1. **If you already suffer dry eyes, has wearing the face mask caused the condition to become worse?**

Much worse

Moderately worse

Slightly worse

I do not have dryness

No

1. **Does wearing a facemask with the eyeglasses hamper your vision, e.g., cause fogging?**

Yes

No

I do not wear eyeglasses

1. **Do you think that during COVID-19 pandemic you become more exposed to screens and electronic devices?**

Yes

No

1. **Select the time spent looking at the screens (mobile, laptop, iPad, or any electronic device) per day during the last few months?**

Less than 2 hours/day

2-4 hours/day

4-6hours/day

More than 6 hours/day
