# Supplementary material for: Is wearing a face mask associated with symptomatic dry eye disease among medical students during the COVID-19 era? An online survey
Source: BMC Ophthalmol. 2022 Apr 7;22:159. doi: 10.1186/s12886-022-02377-z (PMC8988476; doi:10.1186/s12886-022-02377-z)
Supplement: Supplementary file 2 [file 12886_2022_2377_MOESM2_ESM.docx]

Ocular Surface Disease Index© (OSDI©)[2]

Ask your patients the following 12 questions, and circle the number in the box that best represents each answer. Then, fill in boxes A, B, C, D, and E according to the instructions beside each.

Have you experienced any of the following *during the last week?*	All of the time	Most of the time	Half of the time	Some of the time	None of the time
1. Eyes that are sensitive to light? . .	4	3	2	1	0
2. Eyes that feel gritty?	4	3	2	1	0
3. Painful or sore eyes?	4	3	2	1	0
4. Blurred vision?	4	3	2	1	0
5. Poor vision?	4	3	2	1	0

Subtotal score for answers 1 to 5 (A)

Have problems with your eyes limited you in performing any of the following *during the last week?*	All of the time	Most of the time	Half of the time	Some of the time	None of the time	N/A
6. Reading?	4	3	2	1	0	N/A
7. Driving at night?	4	3	2	1	0	N/A
8. Working with a computer or bank machine (ATM)?	4	3	2	1	0	N/A
9. Watching TV?	4	3	2	1	0	N/A

Subtotal score for answers 6 to 9 (B)

Have your eyes felt uncomfortable in any of the following situations *during the last week?*	All of the time	Most of the time	Half of the time	Some of the time	None of the time	N/A
10. Windy conditions?	4	3	2	1	0	N/A
11. Places or areas with low humidity (very dry)?	4	3	2	1	0	N/A
12. Areas that are air conditioned? . . .	4	3	2	1	0	N/A

Subtotal score for answers 10 to 12 (C)
